# Spatial and Temporal Expression Characteristics of the *HBB* Gene Family in Six Different Pig Breeds

**DOI:** 10.3390/genes13101822

**Published:** 2022-10-09

**Authors:** Xin Guo, Zhiguo Liu, Yulian Mu, Lei Huang, Kui Li, Jing Zhang

**Affiliations:** 1 Laboratory of Genetic Breeding, Reproduction and Precision Livestock Farming, School of Animal Science and Nutritional Engineering, Wuhan Polytechnic University, Wuhan 430023, China; 2 Institute of Animal Sciences, Chinese Academy of Agricultural Sciences, Beijing 100193, China; 3 Genome Analysis Laboratory of the Ministry of Agriculture and Rural Affairs, Agricultural Genomics Institute at Shenzhen, Chinese Academy of Agricultural Sciences, Shenzhen 518124, China

**Keywords:** β-thalassemia, pigs, *HBB* gene family, hemoglobin

## Abstract

**Simple Summary:**

β-Thalassemia is one of the most prevalent inherited diseases in China. It is important to develop animal models to accurately simulate human β-thalassemia and there are unique advantages to studying β-thalassemia in pigs. However, there are only few reports on the systematic analysis of the β-thalassemia-related genes and their expression pattern in pigs so far. Therefore, in this study, we firstly predicted 11 porcine hemoglobin-encoding genes and found that there was no *HBG* gene in pigs, indicating that the globin switches might not exist in pigs. A new hemoglobin-encoding gene, ‘*HBB-like*’, was found in pigs, which showed high conservation in its amino sequences between pigs and humans. Then, we studied the evolutionary relationship of hemoglobin-encoding genes in human, pig and mouse. The results showed that the β-chain structure of pig and human was highly similar. In addition, we analyzed the hemoglobin-encoding gene expressions by using the iswine database and qPCR. Our results showed significant differences in the spatiotemporal expression patterns among the four genes (*HBA*, *HBB*, *HBB-like* and *HBE*) in three developmental stages of six different pig breeds. Our study provides an important theoretical basis for further construction of a gene-edited β-thalassemia miniature pig model to assess β--thalassemia treatments.

**Abstract:**

β-Thalassemia induces hemolytic anemia caused by mutations in the β-chain gene locus. As humans progress from embryo to adulthood, hemoglobin recombines twice. To test whether similar hemoglobin reassembly occurs in pigs, bioinformatics tools were used to predict the pig hemoglobin-encoding gene. We then systematically analyzed the expression patterns of the *HBB* gene family in three developmental stages (weaning, sexual maturity and physical maturity) of six different pig breeds (Landrace, Yorkshire, Wuzhishan, Songliao black, Meishan and Tibetan). The results showed that the new hemoglobin coding gene ‘*HBB-like*’ was found in pigs, while the *HBG* gene did not exist in pigs, indicating that human-like reassembly might not exist in pigs. The *HBB* and *HBB-like* genes shared highly similar amino acid sequences and gene sequences. The genes on the β-chain were highly similar between humans and pigs and the amino acid sequences of human and pig *HBB* genes at position 26 and positions 41–42 were identical. qPCR results showed that there were significant differences in the spatiotemporal expression patterns of the four genes (*HBA*, *HBB*, *HBB-like* and *HBE*) across breeds. Our results provide a foundation for follow-up studies assessing the relationship between the gene-encoding hemoglobin and β-thalassemia disease, as well as the construction of a gene-edited β-thalassemia miniature pig model to assess β-thalassemia treatments.

## 1. Introduction

Thalassemias are common autosomal recessive disorders, especially in populations in the Mediterranean region, the Indian subcontinent, Southeast Asia and West Africa [1,2]. Although there are many forms of thalassemia, β-thalassemia is the most common form, characterized by microcytosis and hypochromic anemia, resulting from mutations in the human β-globin locus [3,4]. Nearly 80 to 90 million with minor β-thalassemia and 60–70 thousand affected infants are born annually worldwide [5]. Haemoglobins (Hb) have a tetrameric structure, consisting of two α-like (α or ζ) and two β-like (ε, γ, δ or β) globin chains, each linked to a heme group [6]. Normal hemoglobins include Hb Portland (ζ2γ2), Hb Gower 1 (ζ2ε2), Hb Gower 2 (α2ε2), HbF (α2γ2), HbA1 (α2β2) and HbA2 (α2δ2) [7]. As an individual develops, the globin subunits making up hemoglobin undergo reassembly during switching [8]. The erythrocytes in an embryo contain Hb Portland, Hb Gower 1 and Hb Gower 2; the erythrocytes in a fetus contain predominantly fetal HbF; the erythrocytes in adults contain two adult HbA1 and HbA2 [9,10].

The genes involved in forming hemoglobin at the α-globin locus are *ζ*-globin gene (*HBZ*) and *α*-globin gene (*HBA*). The *β*-globin gene locus includes *ε*-globin gene (*HBE*), *Gɤ*-globin gene (*HBG2*), *Aɤ*-globin gene (*HBG1*), *δ*-globin gene (*HBD*) and *β*-globin gene (*HBB*) [11]. The β-globin genes (i.e., fetal *ɤ*-globin and adult *β*-globin) are expressed in a perfectly tuned way only at specific developmental stages. There are two globin switches in humans, which are HBE to HBG1/HBG2, occurring in the transition from primitive to definitive erythropoiesis and HBG1/HBG2 to HBB occurring in definitive erythropoiesis around the time of birth [12].

To date, more than 900 β-globin variants have been recorded worldwide. These gene alterations often cause either a decrease in β-globin synthesis or completely block synthesis [13]. For example, HbE/β-thalassemia is a G→A substitution at the 26 position of the β-globin gene and replaces Glu with Lys, resulting in a decrease in the β-globin chain [14,15,16,17]. As such, 1–3% of the people in Southern China carry a β-thalassemia allele and over 20 β-thalassemia mutations have been reported in the Chinese population [18]. The most common type of β-thalassemia gene mutation in Southern China is CD41–42 (−CTTT), which accounts for approximately 40% of total β-thalassemia mutations in China. It is caused by the deletion of 4-bp in the *HBB* gene due to a frameshift mutation [19,20]. With widespread population migration, β-thalassemia diseases present an increasing challenge to health services in developing countries [21,22].

Animal models recapitulating both the phenotype and genotype of human disease are valuable in the exploration of pathophysiology and for in vivo evaluation of novel therapeutic treatments. Therefore, it is important to develop animal models to accurately simulate human β-thalassemia for gene therapy or for other more effective therapies. Pig is the optimal experimental animal model for performing clinical research on human disease [23,24,25,26]. Moreover, pigs have unique advantages in studying β-thalassemia and other related blood diseases, compared with mice and rabbits [27,28]. However, there are only few reports on the systematic analysis of the β-thalassemia-related genes and their expression pattern in pigs so far. In this study, we used bioinformatics tools to predict the pig hemoglobin-encoding genes to test whether similar hemoglobin reassembly occurs in pigs. We also systematically analyzed the spatiotemporal expression patterns of the four genes (*HBA*, *HBB*, *HBB-like* and *HBE*) in three developmental stages (weaning, sexual maturity and physical maturity) of six different pig breeds (Landrace, Yorkshire, Wuzhishan, Songliao black, Meishan and Tibetan). Our results will provide a foundation for further studies assessing the relationship between the gene-encoding pig hemoglobin and β-thalassemia disease, as well as the construction of a gene-edited β-thalassemia miniature pig model to assess β-thalassemia treatments.

## 2. Materials and Methods

### 2.1. The Identification of Hemoglobin-Encoding Genes in Pigs and Mice

We downloaded the human hemoglobin-encoding genes sequences from NCBI database to search against the hemoglobin-encoding gene sequences in pig and mouse using the BLASTP program with an e-value of 1 × 10^−^^50^ as the threshold. We preliminarily identified the hemoglobin-encoding genes in pig and mouse by analyzing the results from HMM and BLASTP. We then used the NCBI-CDD web server (http://www.ncbi.nlm.nih.gov/Struc-ture/cdd/wrpsb.cgi, accessed on 23 November 2021) to confirm the prediction [29].

### 2.2. Evolutionary Analysis and Motif Prediction of Human, Mouse and Pig Hemoglobin-Encoding Genes

The amino acid sequences of the hemoglobin-encoding genes in human, pig and mouse were downloaded from the NCBI website. Multiple alignments of the amino acid sequences were performed using ClustalW [30]. The phylogenetic analysis was constructed based on the amino acid sequences of the hemoglobin-encoding genes form human, pig and mouse using a neighbor-joining (NJ) method with 1000 bootstrap replicates and visualized with MEGA5 software. Protein motifs were predicted by using Multiple Em for Motif Elicitation (MEME) (https://meme-suite.org/meme/tools/meme, accessed on 27 April 2022) and protein domain functions were analyzed from Uniprot (https://www.uniprot.org/, accessed on 27 April 2022).

### 2.3. Primer Design

The primers were designed using Primer 5.0 software (Palo Alto, CA, USA) and were synthesized by Sangon Bioengineering Co., Ltd. (Shanghai, China) (Table 1). The forward primer (*HBB-2F*) for the quantitative detection of the *HBB* copy number did not bind to the *HBB-like* gene.

### 2.4. Reverse Transcription Quantitative Real-Time PCR (RT-qPCR)

RT-qPCR analysis of the four genes (*HBB*, *HBB-like*, *HBE* and *HBA*) in five tissues (heart, liver, spleen, lung and kidney) of six pig breeds (Landrace, Yorkshire, Wuzhishang, Songliao black, Meishang, Tibetan) at different development stages was performed. Total RNA was extracted using an RNA extraction kit (MagaBio plus, Hangzhou, Chian). cDNA was synthesized using a RevertAid First Strand cDNA Synthesis Kit (Thermo Scientific, USA). qPCR was performed on an ABI 7500 machine using the SYBR Premix Ex Taq Kit (Vazyme, Nanjing, China) and *18S* ribosomal ribonucleic acid (*18S* rRNA) was used as an endogenous control gene. Relative mRNA expression levels were calculated using the Lg2^-ΔΔCt^ method [31]; the 2^−∆∆Ct^ method was reviewed in [32].

### 2.5. Statistics

The experimental data were analyzed using Prism software (Graphpad prism 8.0). Multiple comparisons of data from multiple groups were performed using analysis of variance (ANOVA). The data are presented as the mean ± standard error of mean (SEM). The level of significance is *p* < 0.05.

## 3. Results

### 3.1. Identification of the Pig Hemoglobin-Encoding Gene Family

In this study, a total of 11 pig hemoglobin-encoding genes and 10 mouse hemoglobin-encoding genes were obtained. Genome-wide hemoglobin-encoding genes in humans, pigs and mice were listed in Table 2. Human hemoglobin-encoding genes were located on chromosomes 11 and 16, while pig hemoglobin-encoding genes were mainly located on chromosomes 9 and 3 and mouse hemoglobin-encoding genes were located on chromosomes 7 and 11. In the prediction of the pig *HBB* gene family, the *HBB-like* gene was also found on chromosome 9. In the NCBI database, the three genes on pig chromosome 9 were sequenced from upstream to downstream as *HBE*, the *HBB-like* and *HBB* gene. The four genes on pig chromosome 3 were sequenced from upstream to downstream as *HBZ*, *HBM*, *HBA* and *HBQ* gene (date were not shown). No new gene *HBG*(*γ*) was found and there might not be a double switch similar in pigs to that in humans on the β-globin chain. No *HBD* and *HBM* genes were predicted in mice.

### 3.2. Evolutionary Analysis of Human, Pig and Mouse Hemoglobin-Encoding Genes

**Figure 1:**
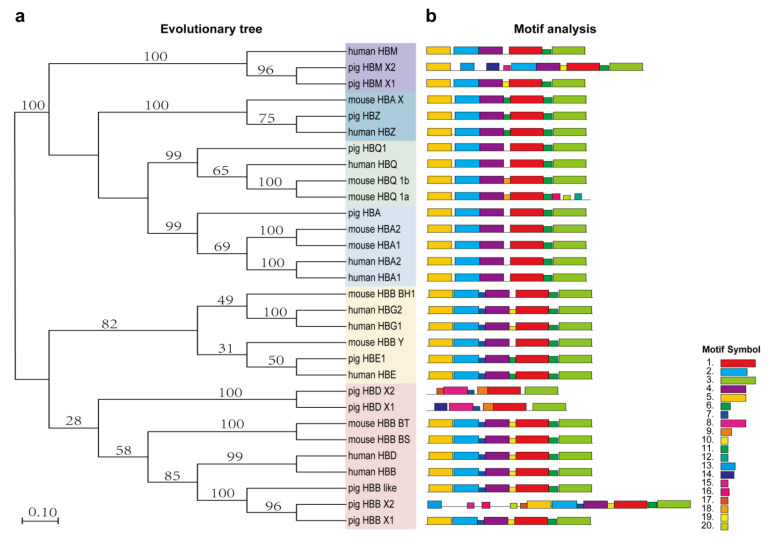
Phylogenetic relationships and motif composition of human, mouse and pig hemoglobin-encoding genes. (**a**) Represents the phylogenetic tree constructed by MEGA 5.0 software and different gene colors represent different clades; (**b**) represents the motifs constructed from human, mouse and pig protein sequences. Different colored boxes represent different motifs.

**Figure 2:**
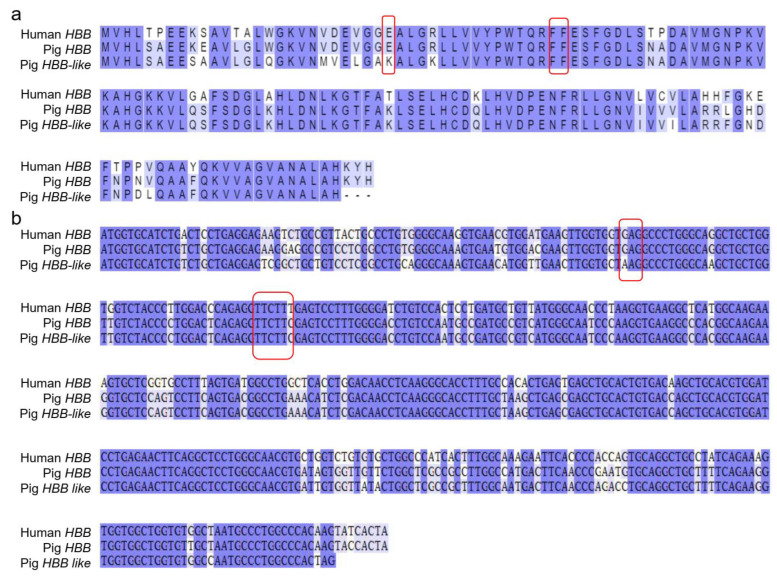
Sequence alignment of human *HBB* gene, pig *HBB* gene and pig *HBB-like* gene. (**a**) Sequence alignment of human *HBB*, pig *HBB* and *HBB-like* proteins; (**b**) The CDS sequence alignment of human *HBB* gene, pig *HBB* gene and pig *HBB-like* gene.

To analyze the evolutionary relationship of hemoglobin-encoding genes in human, pig and mouse, a phylogenetic tree was constructed using amino acid sequences. A total of 10 human sequences, 11 pig sequences and 10 mouse sequences of hemoglobin-encoding genes were evaluated in the phylogenetic tree (Figure 1a). The hemoglobin-encoding genes were divided into two outgroups, α-globin and β-globin. Among them, the mouse *HBB*-*BH2* gene was the LCR of the mouse 5′ regulatory region and were not displayed in the phylogenetic tree. From an evolutionary point of view, *HBM* and *HBZ* on the pig α-globin chain and *HBE* on the β-globin chain were more similar to humans. Human *HBB* and *HBD* genes were evolutionally closely related to the pig *HBB* gene but were distantly related to pig *HBD* gene. According to the MEM motif, we analyzed and constructed a schematic diagram of the coding hemoglobin structure of pig, mouse and human (Figure 1b; Supplementary Figure S1) and identified a total of 20 different conserved motifs (Figure 1). Most coding genes contained the first six motifs, while the pig *HBD* gene lacked motif 2 and motif 5, which were not involved in the expression of the hemoglobin gene. In addition, the *HBB-like* gene was similar to that of humans in the phylogenetic tree (Figure 1a) and the motifs were similar to the *HBB* structure in pig and human (Figure 2b).

### 3.3. HBB and HBB-like Gene Sequence Analysis

The CDS sequences and protein sequences of human *HBB* gene, porcine *HBB* gene and *HBB-like* gene were downloaded from the NCBI website and compared by Blast through the EMBL-EBI online website. The results demonstrated that the pig HBB and human HBB proteins had the same length and were highly conserved with 85.03% amino acid sequence identity. Especially in the mutant regions (Codon41–42 and Codon26), their amino acid was identical (Figure 2a). Pig and human *HBB* gene CDS sequences had high alignment similarity (percent identity, 84.68%), with only one base difference in the coding sequences at the Codon41-42, while the human and pig *HBB* gene had the same coding sequence at the Codon26 locus (Figure 2b). The protein sequence encoded by *HBB-like* gene was highly similar to *HBB* gene in pig, suggesting the two genes might have similar biological functions (Figure 2a). The 30bp upstream and 200bp downstream sequences of Codon 41–42 sequences in CDS of pig *HBB* and *HBB-like* genes were completely identical (Figure 2b). Analysis of the Ensembl database demonstrated that their 250 bp genome sequences upstream and downstream of Codon41–42 were almost identical (percent identity, 96.77%).

### 3.4. Expression Levels of the HBB Gene Family in Different Tissues

**Figure 3:**
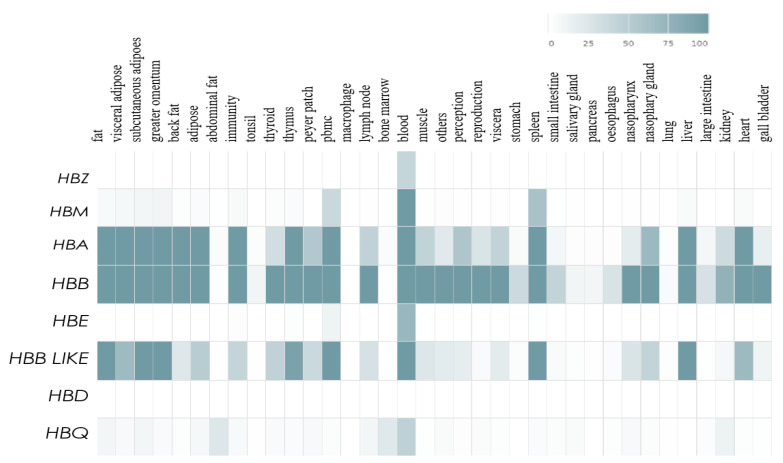
Expression patterns of the *eight porcine hemoglobin-encoding genes* in the iswine database.

We analyzed the expression information of eight porcine hemoglobin-encoding genes (*HBZ*, *HBM*, *HBA*, *HBB*, *HBE*, *HBB-like*, *HBD* and *HBQ*) in the iswine database (http://iswine.iomics.pro, accessed on 27 April 2022). A tissue expression heatmap of the eight genes was shown in Figure 3. According to the data results, *HBA*, *HBB* and *HBB-like* genes were abundantly expressed in multiple tissues, such as adipose tissue, greater omentum, thymus, blood tissue, spleen, liver and heart. However, *HBB* and *HBB-like* genes showed obviously different expression levels in some tissues, such as back fat, lymph node, muscle and thymus. While, *HBZ*, *HBM* and *HBE* genes were only expressed in a few tissues according to the iswine database. In addition, there was no tissue expression profile information of *HBD* gene in the iswine database.

### 3.5. Spatiotemporal Expression Patterns of Hemoglobin-Encoding Genes in Adult Pigs

**Figure 4:**
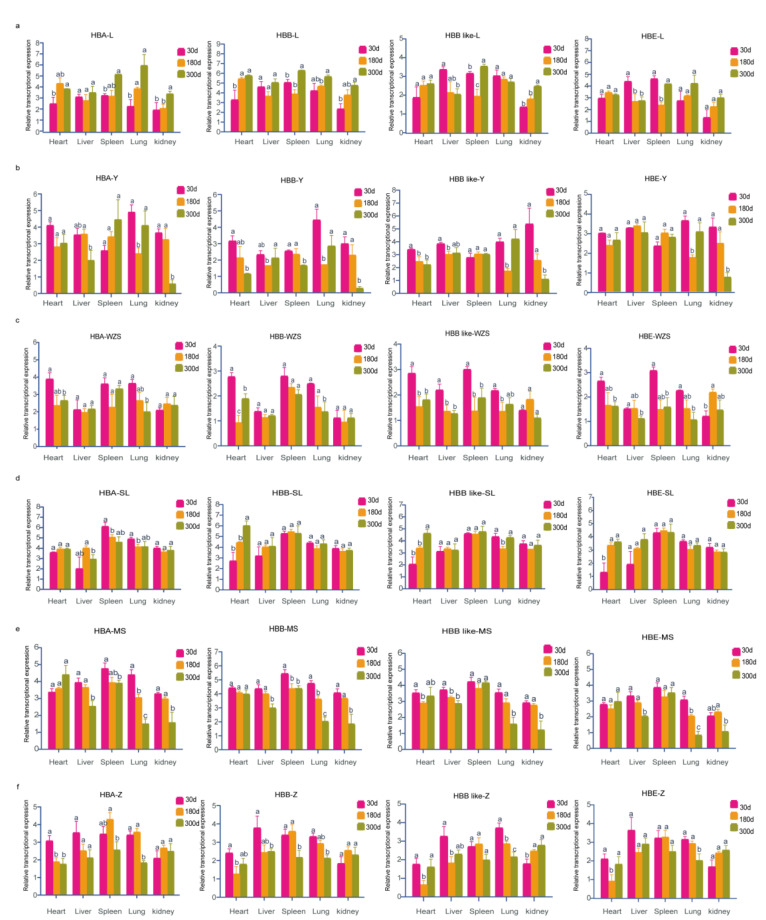
Expression patterns of hemoglobin-encoding genes in adult pig different tissues. (**a**–**f**) represent expression of *HBA*, *HBB*, the *HBB-like* and *HBE* gene in Landrace (L), Yorkshire (Y), Wuzhishan (WZS), Songliao black (SL), Meishan (MS) and Tibetan (Z), respectively. Data are shown as mean ± SEM (n = 3 to 6), student’s *t*-test. Relative expression was calculated as: Lg2^-ΔΔCt^. *p* < 0.05 was considered to be statistically significant. significant a, b, c indicate significant difference.

We selected three time points (30 d, 180 d and 300 d) to detect the four gene expressions in different tissues (heart, liver, spleen, lung and kidney) of six different pig breeds. The results were shown in Figure 4. Gene expression results showed that *HBA* and *HBB* genes were up-regulated in the heart, spleen, lung and kidney tissues derived from the Landrace pig, while *HBE* and *HBB-like* genes were down-regulated in liver with the increase in age. In addition, the expressions of *HBE* and *HBB-like* genes were significantly lower at 180 days than at any other age in spleen tissue of the Landrace pig. In heart tissue of Yorkshire pigs, the expression levels of *HBB* and *HBB-like* genes were down-regulated with the increase in age. In lung tissue of Yorkshire, the expressions of the four genes had no significant differences between 30 days and 300 days and the expression levels were significantly higher than that at 180 days. In kidney tissue, the expression levels of the four genes decreased with the increase in age and reached the lowest level at 300 days. In Wuzhishan pigs, *HBA* and *HBB* genes were down-regulated with the increase in age in heart and lung tissues, while *HBE* and *HBB-like* genes were down-regulated with age in heart, liver, spleen and lung tissues. In Songliao Black pigs, the expressions of *HBB*, *HBE* and *HBB-like* genes increased in the heart tissue. In heart tissue of the Meishan pigs, there were no significant differences in the expressions of *HBA*, *HBB* and *HBE* genes in the three periods, but the expression of *HBB-like* gene decreased at first but then increased with the increase in age. In liver, lung and kidney tissues from the Meishan pigs, the four genes were significantly down-regulated at 300 days of age. Moreover, the expressions of *HBA* and *HBB* genes decreased with the increase in age, but the expressions of *HBB-like* and *HBE* genes had no significant difference in spleen tissue form the Meishan pigs. In heart tissue of the Tibetan pigs, the expression of *HBA* gene decreased at 180 and 300 days, but the other three genes were only deceased at 180 days. In the Tibetan pig lung tissue, the expression levels of the four genes showed a significant decrease at 300 days of age.

## 4. Discussion

There are many kinds of mutations in β-thalassemia. The most common type of thalassemia in Asia is the HbE/β-type, while the most common mutation in China is in Codons 41/42 (−CTTT). These two β-thalassemia mutations both occur in the *HBB* gene [33,34]. Because reproductive cycle in pig is relatively short and pigs have similar metabolism and immunity to humans, it is important to perform disease-related genetic research on pigs. In this study, we predicted 10 and 11 hemoglobin-encoding genes in mouse and pig according to human hemoglobin-encoding genes sequences, respectively, and constructed a phylogenetic tree to analyze their conserved domains. Our results showed that the conserved regions of the β-chain in pigs were highly similar except for the *HBD* gene, while the *HBE* genes of humans and pigs were more closely related and the consistency of the conserved regions was high. The two transitions from embryonic to postnatal globin exist in human [35]. The absence of the pig hemoglobin-encoding gene *HBG* indicated that the two transitions might not exist in pigs. In addition, a new *HBB-like* gene was discovered in the gene-encoding pig hemoglobin. A comparison of protein and gene sequences among human and pig *HBB* genes and pig *HBB-like* gene demonstrated that their protein sequences had similar lengths and high similarity. In humans and pigs, Codons 41/42 (−CTTT) differed by only one base in the *HBB* gene, while Codons 26 were identical. These results suggested that a β-thalassemia disease model could be constructed around the *HBB* gene in pigs.

Due to the presence of *HBF* expression during the fetal period, there are no obvious symptoms of anemia at birth, but by six months of age, if the synthesis of β chains continues to decrease, symptoms of thalassemia will appear [36,37]. Therefore, in this study, we detected the key gene (*HBA*, *HBB*, *HBB-like* and *HBE*) expressions in some tissues (heart, liver, spleen, lung and kidney) of different pig breeds at 30, 180 and 300 days of age. We found that the temporal and spatial expression patterns of these genes were significantly different in various tissues of the six pig breeds. For example, the expressions of *HBA*, *HBB* and *HBB-like* gene increased in the kidney of Landrace pigs at 300 days of age but decreased in the kidney of Yorkshire and Meishan pigs. However, the expression patterns of these genes in different tissues of the same pig species were similar. Interestingly, the expression of *HBB* and *HBB-like* genes were not consistent in different pig breeds and different tissues, suggesting that the *HBB-like* gene might have other potential functions. Next, we will perform functional detection experiments around the *HBB-like* gene.

## 5. Conclusions

In this study, we predicted 11 hemoglobin-encoding genes in pigs. According to our prediction results, we further constructed a phylogenetic tree to analyze their conserved domains among humans, mice and pigs. Our results showed that the β-chain structures between pigs and humans were highly similar. Interestingly, we did not find a porcine *HBG* gene, indicating that the globin switches might not exist in pigs. A new hemoglobin-encoding gene, *‘HBB-like’,* was found in pigs, with its encoding protein sequence sharing high similarity with the *HBB* gene. Nevertheless, the spatiotemporal expression patterns of *HBB* and *HBB-like* genes showed significant difference across breeds. Together, our data provide an insight into the basic reference for construction of a gene-edited β-thalassemia pig model in the future.

## Figures and Tables

**Table 1:** *HBB* gene family primer sequences.

Primers	Sequence (5′–3′)	Product Length, bp
*SUS-Qhbb1-F*	aatgtggacgaagttggtggt	270
*SUS-Qhbb1-R*	gttgcccaggagcctgaagt	
*SUS-HBB-like-F*	tcggctgctgtcctcggcctgca	303
*SUS-HBB-like-R*	gttgcccaggagcctgaagt	
*SUS-HBE1-F*	tcctggtggtctacccttgg	233
*SUS-HBE1-R*	gttgcccaggagcctgaagt	
*SUS-HBA-F*	agaggccctggaaagaatgt	278
*SUS-HBA-R*	ggttgaaatcatcggggtgg	
*18S-F*	gtaacccgttgaaccccatt	151
*18S-R*	ccatccaatcggtagtagcg	

**Table 2:** Human, pig and mouse hemoglobin-encoding genes.

Species	Gene Symbol	Protein ID	Chromosome
*Homo sapiens*	*HBB*	ENST00000335295	11
	*HBD*	ENST00000292901	11
	*HBE1*	ENST00000396895	11
	*HBG1*	ENST00000330597	11
	*HBG2*	ENST00000336906	11
	*HBA1*	ENST00000320868	16
	*HBA2*	ENST00000251595	16
	*HBM*	ENST00000356815	16
	*HBQ1*	ENST00000199708	16
	*HBZ*	ENST00000252951	16
*Sus scrofa*	*HBB X1*	ENSSSCP00000067521	9
	*HBB X2*	ENSSSCP00015021905	9
	*HBB-like*	ENSSSCP00000015649	9
	*HBD X1*	ENSSSCP00000059042	9
	*HBD X2*	ENSSSCP00000045757	9
	*HBE1*	ENSSSCP00000015648	9
	*HBA*	ENSSSCP00000028944	3
	*HBM X1*	ENSSSCP00000063903	3
	*HBM X2*	ENSSSCP00000008516	3
	*HBQ1*	ENSSSCP00000029523	3
	*HBZ*	ENSSSCP00000008514	3
*Mus Musculus*	*HBA-x*	ENSMUSP00000020531	11
	*HBQ 1a*	ENSMUSP00000020535	11
	*HBQ 1b*	ENSMUSP00000098936	11
	*HBA a1*	ENSMUSP00000090897	11
	*HBA a2*	ENSMUSP00000090895	11
	*HBB bs*	ENSMUSP00000023934	7
	*HBB y*	ENSMUSP00000033229	7
	*HBB bh1*	ENSMUSP00000064865	7
	*HBB bt*	ENSMUSP00000095794	7
	*HBB bh2*	ENSMUSP00000102479	7

## Data Availability

The data that support the findings of this study are available from the corresponding author upon reasonable request.

## Supplementary Materials

The following supporting information can be downloaded at: https://www.mdpi.com/article/10.3390/genes13101822/s1. Supplementary Figure S1. Analysis and distribution of conserved motifs in human, pig and mouse hemoglobin-encoding genes.
