# Supplementary material for: Spatial and Temporal Expression Characteristics of the HBB Gene Family in Six Different Pig Breeds
Source: Genes (Basel). 2022 Oct 9;13(10):1822. doi: 10.3390/genes13101822 (PMC9601290; doi:10.3390/genes13101822)
Supplement: Supplementary file 1 [file genes-13-01822-s001.zip › Supplementary Figure S1.pdf]

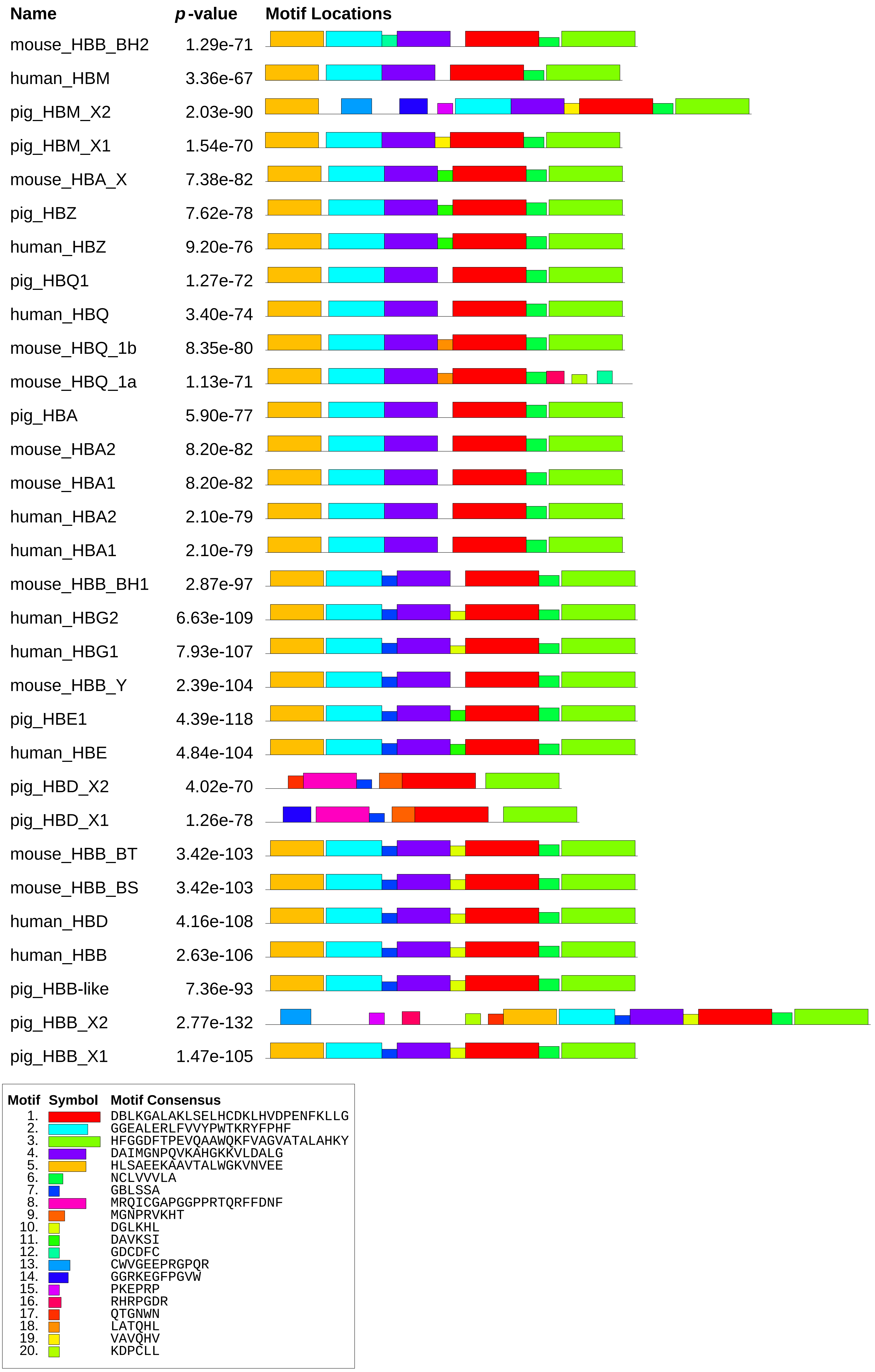

Name
p-value
Motif Locations
mouse_HBB_BH2 1.29e-71
human_HBM 3.36e-67
pig_HBM_X2 2.03e-90
pig_HBM_X1 1.54e-70
mouse_HBA_X 7.38e-82
pig_HBZ 7.62e-78
human_HBZ 9.20e-76
pig_HBQ1 1.27e-72
human_HBQ 3.40e-74
mouse_HBQ_1b 8.35e-80
mouse_HBQ_1a 1.13e-71
pig_HBA 5.90e-77
mouse_HBA2 8.20e-82
mouse_HBA1 8.20e-82
human_HBA2 2.10e-79
human_HBA1 2.10e-79
mouse_HBB_BH1 2.87e-97
human_HBG2 6.63e-109
human_HBG1 7.93e-107
mouse_HBB_Y 2.39e-104
pig_HBE1 4.39e-118
human_HBE 4.84e-104
pig_HBD_X2 4.02e-70
pig_HBD_X1 1.26e-78
mouse_HBB_BT 3.42e-103
mouse_HBB_BS 3.42e-103
human_HBD 4.16e-108
human_HBB 2.63e-106
pig_HBB-like 7.36e-93
pig_HBB_X2 2.77e-132
pig_HBB_X1 1.47e-105
Motif Symbol Motif Consensus
1. DBLKGALAKLSELHCDKLHVDPENFKLLG
2. GGEALERLFVVYPWTKRYFPHF
3. HFGGDFTPEVQAAWQKFVAGVATALAHKY
4. DAIMGNPQVKAHGKKVLDALG
5. HLSAEEKAAVTALWGKVNVEE
6. NCLVVVLA
7. GBLSSA
8. MRQICGAPGGPPRTQRFFDNF
9. MGNPRVKHT
10. DGLKHL
11. DAVKSI
12. GDCDFC
13. CWVGEEPRGPQR
14. GGRKEGFPGVW
15. PKEPRP
16. RHRPGDR
17. QTGNWN
18. LATQHL
19. VAVQHV
20. KDPCLL
